# The Effect of Increasing the Amount of Indium Alloying Material on the Efficiency of Sacrificial Aluminium Anodes

**DOI:** 10.3390/ma14071755

**Published:** 2021-04-02

**Authors:** Krzysztof Zakowski, Juliusz Orlikowski, Kazimierz Darowicki, Marcin Czekajlo, Piotr Iglinski, Kinga Domanska

**Affiliations:** 1 Department of Electrochemistry, Corrosion and Materials Engineering, Faculty of Chemistry, Gdansk University of Technology, 11/12 Gabriela Narutowicza Street, 80-233 Gdansk, Poland; juliusz.orlikowski@pg.edu.pl (J.O.); kazimierz.darowicki@pg.edu.pl (K.D.); 2 KGHM Polska Miedz S.A. Ore Concentration Plant, 1 Kopalniana Street, 59-101 Polkowice, Poland; marcin.czekajlo@kghm.com; 3 LOTOS Petrobaltic Ltd., 9 Stary Dwor Street, 80-758 Gdansk, Poland; piotr.iglinski@lotospetrobaltic.pl (P.I.); kinga.domanska@lotospetrobaltic.pl (K.D.)

**Keywords:** Al-Zn-In, aluminium sacrificial anode, aluminium galvanic anode, cathodic protection, current capacity of sacrificial anode, closed circuit potential of sacrificial anode

## Abstract

Al-Zn-In alloys having 4.2% zinc content and various indium content in the range of 0.02–0.2% were tested with respect to the most important electrochemical properties of sacrificial anodes in a cathodic protection, i.e., the current capacity and potential of the operating anode. The distribution of In and Zn in the tested alloys was mapped by means of the EDX technique, which demonstrated that these elements dissolve well in the alloy matrix and are evenly distributed within it. The current capacity of such alloys was determined by means of the method of determining the mass loss during the dissolution by a current of known charge. The results obtained demonstrate that the current capacity of Al-Zn-In alloy decreases with the increase in the In content, which results in an increased consumption of anode material and shorter lifetime of anodes. With 0.02% In content, the capacity amounted to approx. 2500 Ah/kg, whereas the alloy with 0.2% In had as much as 30% lower capacity amounting to approx. 1750 Ah/kg. Microscopic examination for the morphology and surface profile of the samples after their exposure demonstrated that a higher indium content in the alloy results in a more uneven general corrosion pattern during the dissolution of such alloy, and the cavities (pits) appearing on the alloy surface are larger and deeper. As the indium content is increased from 0.02% to 0.05%, the Al-Zn-In alloy potential decreases by about 50 mV to −1100 mV vs. Ag/AgCl electrode, which is advantageous in terms of using this alloy as a sacrificial anode. When the indium content is further increased from 0.05% to 0.2%, the potential of the alloy is no longer changed to a more negative one. The results obtained from all these tests demonstrate that alloys containing up to 0.05% of In additive are practically applicable for cathodic protection.

## 1. Introduction

Aluminium is the third most abundant element (after oxygen and silicon) on Earth. Aluminium products are used as semi-finished or finished products in practically every industry, e.g., automotive, packaging, construction, chemical, energy, electronics, metallurgy, etc. 

Aluminium is also used for the cathodic protection, which is one of the main anti-corrosion technologies for underwater and underground steel structures. In the cathodic protection (which is based on lowering the potential of the protected structure by means of the current flowing from anodes), it is necessary to provide an amount of the protective current that polarizes the structure up to the protective potential [1]. Thereafter, the corrosion processes are inhibited and the estimated corrosion rate of the structure is less than 0.01 mm/year [2]. Too low a density of the polarizing current makes the structure polarized up to the potential more positive than the protective potential. In such a case, the effect of partial cathodic protection is obtained in which the corrosion processes are partially inhibited, but not completely. 

Cathodic protection can be implemented by means of two technologies. The first is the ICCP (impressed current cathodic protection system) [3]. The source of the current is a polarizing device—the so-called cathodic protection station supplied from the power grid and cooperating with polarizing anodes which are usually made of non-soluble materials (e.g., titanium and ruthenium coated with MMO—mixed metal oxide [4,5]) or hardly soluble materials (e.g., cast iron [6]). The main advantage of ICCP is the possibility of obtaining a high intensity protection current from anodes, due to a possible high output voltage from the device, which amounts up to 50 V for underground structures and up to 24 V for offshore structures. Hence, a small number of anodes necessary to polarize the entire surface of the protected structure will be sufficient [7].

The second cathodic protection technology is an application of sacrificial anodes (galvanic anodes) directly connected to the protected structure [8,9]. These anodes are made of metals with a more negative potential than the protected structure, which constitute a galvanic cell altogether with the structure. In this cell, anodes are dissolved and supply the cathodic protection current which flows from anodes through the electrolyte (water, earth) to the protected structure. Offshore steel structures are usually protected with aluminium or zinc anodes. Magnesium anodes are used to protect underground structures, although it is also possible to use them to protect offshore structures [10,11]. The potential difference between the operating sacrificial anode and the protected steel structure is small, only approx. 0.25 V, thus the current intensity from the galvanic anode is relatively small. Therefore, a large number of sacrificial anodes is required for offshore structures in order to provide the required amount of cathodic protection current, particularly when the barrier properties of the structure’s protective coating are poor [12,13]. 

A particular feature of sacrificial anodes is the fact they are dissolved during their operation and it is periodically necessary to replace them with new ones. Another related aspect is the possibility of contaminating the natural environment with anode dissolution products. Research in this field demonstrated that within the area of operating anodes the concentration of aluminium or zinc in seawater increases only slightly; on the other hand, it is high in sea mud in the direct neighbourhood of operating anodes [14,15].

An oxide layer is formed on pure aluminium, which has a passivating effect [16]. A particular improvement in the electrochemical properties of aluminium anodes is achieved by means of the respective alloying additives. The purpose of particular additives is to activate the passivated Al surface, the purpose of other additives is to lower the anode alloy’s potential, whereas the purpose of further additives is to increase the current efficiency [17]. 

Zn is primarily used for the activation of the passivated aluminium surface, and it is usually added in the amount of 1–5% [18]. The alloying additives such as In, Cd, Ga, Sn, Hg, Mg are also beneficial, because they act as aluminium depasivators [19]. On the other hand, when the amount of Sn is increased above 0.2%, similarly to Mg above 1%, the dissolution rate of aluminium anodes is adversely increased [20]. Zn and In change the anode potential to a more negative one and improve the microstructural uniformity of anodes—the latter leads to more uniform corrosion [21]. Cu alloying additive, which is added to increase the current efficiency, unfortunately leads to the Al_2_Cu intermetallic phase at grain boundaries, which results in the uneven anode dissolution [22]. Therefore, Sm is added in order to modify the shape of Al_2_Cu, making it finer and making the corrosion of Al–Zn–Cu alloy uniform [23]. Pitting and crevice corrosion occurring on the surface of Al–5Zn alloy can be limited by adding 2% Mg, 0.15% Mn and 0.02% In, which is evidenced by microstructure examinations of the corroded alloy surfaces with different contents of these additives [24]. 

The properties of aluminium anodes are adversely affected by impurities, especially Fe and Si, which increase the corrosion rate of Al–Zn alloys and adversely increase the potential of operating anode to a more positive value [25].

As discussed above, the scientific literature describes the influence of In on the activation of the passivated surface of aluminium alloys, on the reduction in their self-corrosion and on the OCP value (open circuit potential, which is the corrosive potential of non-operating anode). In terms of the efficiency and operation of the cathodic protection systems, the influence of this element on other properties is very important, particularly the influence on the anode alloy efficiency, its current capacity, as well as electronegative CCP (closed circuit potential) value of the operating anode, despite its dissolution. Large current capacity contributes to the extended lifetime of the anode, i.e., the time it is dissolved, leading to the economic effect. The high electronegative potential of the operating anode enables the polarisation of the protected structure up to the protective potential, even when anodes are covered with corrosion by-products and marine organisms [26,27], or when there is an increased demand for cathodic protection current after the stormy period during which stormy sea water destroys the non-conductive calcareous deposits existing on the protected structure (deposits reduce the demand for cathodic protection current, similarly to the protective coatings [28]).

Murray and Morton [29] examined the electrochemical properties of Al–Zn–In alloys with low In content, in a 3% NaCl solution. The results obtained demonstrated that the alloy with 0.02% In content had the best parameters in terms of the CCP potential and the dissolution rate (which is reciprocal to the current capacity). Similar results were obtained by Keyvani, Saremi and Saeri, who tested Al-Zn-In alloys with 1–6% Zn and 0.01–0.05% In [21].

Research centres around the world are constantly examining the influence of various additives on the properties of galvanic anode alloys applicable for cathodic protection, in order to identify the alloys with the best electrochemical properties and extend the service life of anodes. The purpose of the research in this publication is the determination of the effect of an increased amount of indium, in the range of 0.02–0.20%, on the current capacity and the CCP potential of Al-Zn-In sacrificial anode alloys. These electrochemical properties are very important from a practical point of view for designers and users of cathodic protection installations for marine structures. The novelty of the presented work in comparison with the literature are: (i) a wide range of indium addition amounts was tested (for example, in work [21] the content of 0.01–0.05% was tested), (ii) 3D images were used together with the height profiles and the roughness coefficient of the samples surface to explain the cause of changes in the current capacity of the tested alloys, (iii) a hypothesis explaining the mechanism of an observed behaviour of the alloys depending on the indium content was proposed. 

## 2. Materials

The tests were carried out on the samples of prepared Al-Zn-In alloys containing the same zinc content and a different indium content. It was assumed that the amount of indium in the tested alloys will be in the range of 0.02–0.20%, by weight. The selected zinc content in alloys amounted to 4.2%, which is the average level used in the commercial manufacturing of aluminium anodes applicable for the cathodic protection of offshore structures, where the zinc content is usually in the range of 3.0–5.5%. 

The alloys for tests were prepared as follows: A base alloy was prepared, in which the calculated Zn content amounted to 4.2% and the In content amounted to 0.02%. The alloying elements were melted in an electric laboratory furnace in a silicon carbide crucible. The samples were cast from a portion of the base alloy—these were the samples with the smallest amount of indium. Then, the calculated amounts of indium were added to the base alloy, and samples containing more and more indium were cast from the subsequently obtained alloys. In this way, six alloys were obtained in total, in which the Zn content and impurities were practically constant, while the In content increased. Chemical composition of the samples was determined by means of the emission spectrometer. The content of impurities in the base alloy amounted to: Cd < 0.002%, Si < 0.12%, Fe < 0.08%, Cu < 0.003%. 

The content of various components in the finally obtained alloys, determined by spectrometry, is presented in Table 1. 

The samples for SEM microscopic tests and the samples for electrochemical tests were cut from the obtained alloy ingots. The sample preparation method for particular types of tests is described in the following sections.

## 3. Methods

### 3.1. Methodology of Microscopic Research 

The microscopic tests included: (a) determining the distribution of alloying elements in the tested alloys, (b) examining the morphology of the samples after their electrochemical exposure.

Ad. (a) The distribution of alloying elements in the tested samples of Al-Zn-In alloys was examined by means of the HITACHI S-3400N Scanning Electron Microscope (manufacturer: Hitachi High Technologies America Inc., Schaumburg, IL, USA) and the UltraDry X-ray detector, with the NSS 312 X-ray microanalysis system (manufacturer: Thermo Fisher Scientific, Waltham, MA, USA). The EDS (energy-dispersive X-ray spectroscopy) method was used to map the selected sample area and to present the distribution of elements on the tested sample surface.

**Figure 1:**
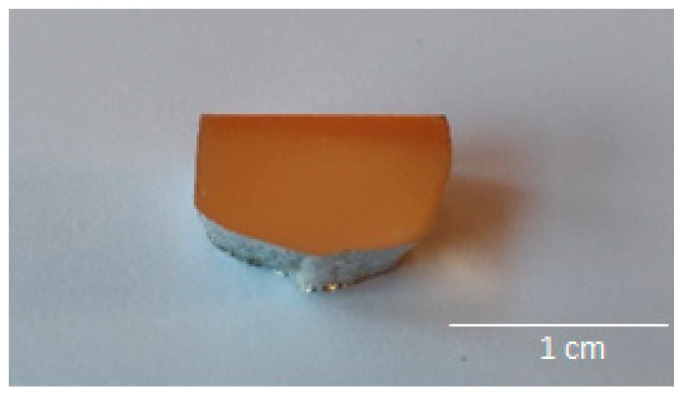
An example sample prepared for SEM tests.

The tested alloy samples with an area of about 1 cm^2^ were prepared for SEM testing by polishing the surface. The samples were polished by 600-grit, 2000-grit and 5000-grit sandpaper and then polished with 3 μm diamond paste. The view of an exemplary sample, which was prepared for testing, is presented in Figure 1.

Ad. (b) The surface morphology of samples after completion of the electrochemical tests was examined by means of the Nikon MA200 metallurgical optical microscope (manufacturer: NIKON CORRPORATION, Tokyo, Japan). In addition to the pictures of the sample surfaces, a computer analysis for the surface profile was also performed, and the roughness coefficient was determined.

### 3.2. Methodology of Electrochemical Research

The current capacity for the prepared alloys was determined by means of the sample mass loss method, which was caused by the outflow of an electric charge of a known value. The method of the 4-day electrochemical test was implemented in accordance with the DNVGL-RP-B401 standard [30]. In this test, the current flow from the anode to the auxiliary steel cathode is forced by a galvanostat. Application of the galvanostat facilitates the application of precisely defined current loads for the anode during tests, with different intensities on individual days, which are much greater than during the normal operation of the anode in cathodic protection installations. Therefore, the test is very “demanding” for the anode material. Various loads on the sacrificial anodes also occur during their operation in real offshore conditions, e.g.,: (i) an increased current demand of the protected structure during its initial cathodic polarisation, from the corrosion potential to the protective potential, (ii) standard working conditions of sacrificial anodes (the so-called operational density of polarising current), (iii) an increased density of polarising current after the stormy period and during the depolarisation of the protected structure, which is related to the destruction of sediments on the structure by a stormy sea.

#### 3.2.1. Method of Preparing the Samples for Electrochemical Tests 

The samples for electrochemical tests were prepared in a cylindrical shape with a diameter of 10 mm and a height of 50 mm. In the base of the cylinder, a hole was drilled and threaded, into which a threaded steel wire with a diameter of 0.5 cm and a length of 20 cm was screwed, which was used to fix the sample in the measuring vessel and to connect the electrical wires to the sample. The wire was secured along its entire length with a plastic heat-shrinkable tubing, in order to prevent it from coming into contact with the electrolyte in the measuring vessel. The connecting point for the wire to the test sample was secured with epoxy resin, which protected it against the penetration of electrolyte into the connecting point and protected the connection against corrosion. 

**Figure 2:**
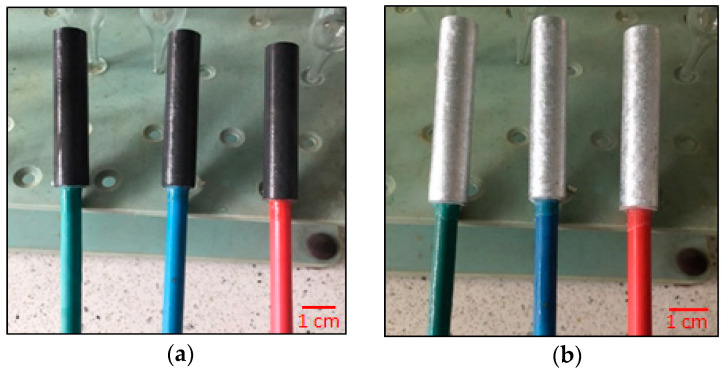
View of the Al-Zn-In alloy sample specimens prepared for electrochemical tests: (**a**) After activation of the sample surfaces in NaOH solution; (**b**) After cleaning the sample surfaces from black deposits in HNO_3_.

Because the surface of the tested samples could passivate during their mechanical processing [31], the sample surface was cleaned of the passive layer according to the NACE TM 0190 standard [32] immediately before the exposure, by immersing the sample for 5 min in a hot (80 °C) sodium hydroxide solution (50 g of NaOH was dissolved in 1 dm^3^ distilled water). In order to remove the black coating formed during the above activity (by-products of chemical reaction between the alloy and NaOH) from the surfaces of the samples, the samples were immersed in a concentrated solution of nitric acid (V) for a few seconds, then rinsed in distilled water and acetone and dried in a stream of hot air for 15 min. After cooling down, the samples were weighed to the nearest 0.1 mg. The appearance of the exemplary samples, prepared for the exposure, is shown in Figure 2.

#### 3.2.2. Measuring System

The samples were exposed in glass vessels with the volume of 5 dm^3^. An auxiliary cathode made of a sheet of S235JR2 carbon steel with a thickness of 0.5 mm was attached in each of them. The sheet was bent into a tube with a diameter of 20 cm and a length of 20 cm, and it was placed next to the vessel wall. The anode sample for testing was placed in the centre, inside the cathode. The Ag/AgCl reference electrode was attached next to the test sample in such a way that the tip of the electrode was at distance of 1 mm from the sample surface. Due to such a close arrangement of the electrode and the anode surface, the measured value of anode potential during its polarisation included a negligible value of the IR ohmic voltage drop. After fixing all the elements, the measuring vessel was filled with electrolyte. An unobstructed air access was provided to the electrolyte. The tests were carried out at room temperature.

**Figure 3:**
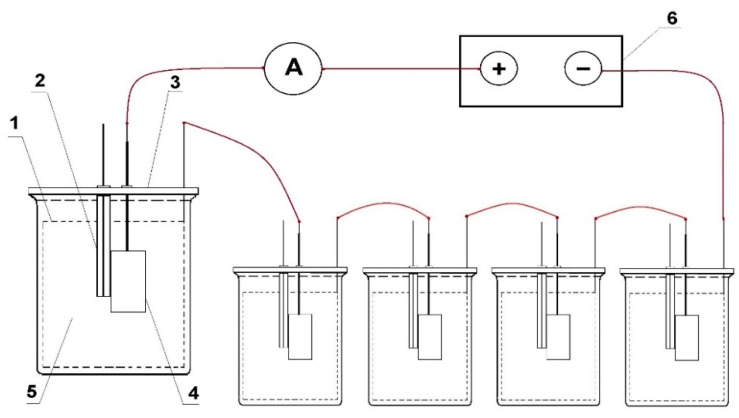
Diagram of the measuring set: 1—auxiliary cathode, 2—Ag/AgCl reference electrode, 3—measuring vessel, 4—sample anode alloy under test, 5—electrolyte, 6—galvanostat.

The source of the polarising current was the galvanostat, type 0931(manufacturer: ATLAS-SOLLICH, Gdansk, Poland). In order to enable the experiment simultaneously in several vessels, the measuring systems were connected with each other by electric cables in a series, in the following manner: output (+) of the galvanostat with the anode in the first vessel, the cathode in the first vessel with the anode in the second vessel, etc., the cathode in the last vessel with the galvanostat output (−). The galvanostat maintained the predetermined current value with an accuracy of ±0.01 mA. A diagram of the measuring set is shown in Figure 3.

#### 3.2.3. Electrolyte

The tests were carried out in synthetic seawater prepared in accordance with ASTM D1141-98 “Standard Practice for the Preparation of Substitute Ocean Water”. Chemical reagents in the amounts specified in Table 2 were used per every 10 dm^3^ of tap water, in order to make the solution.

#### 3.2.4. Current Load on the Samples during the Exposure 

Loading the samples with the current, which flows from them to the auxiliary cathode, was changed every 24 h, in accordance with the methodology specified in DNVGL-RP401 [30]. During the 4-day experiment, the current densities indicated in Table 3 were used. The intensity of the current I_i_ set in the galvanostat was calculated as the product of the required current density j_i_ and area of the sample under the exposure.

#### 3.2.5. Method of Determining the Current Capacity of the Tested Alloys

The current capacity (also known as the electrochemical efficiency) was determined based on the amount of the sample mass loss due to the outflow of an electric charge of known value. 

Before measuring the sample masses after the exposure, corrosion by-products were removed from them according to the NACE TM 0190 [32] standard, by immersing the samples for 10 min in a hot (80 °C) cleansing solution made of 20 g chromium trioxide CrO_3_ and 30 mL phosphoric acid H_3_PO_4_ dissolved in 1 dm^3^ distilled water. The samples were rinsed in tap water and dried in a stream of hot air for 15 min after removing them from the cleansing solution. After cooling down, the samples were weighed to the nearest 0.1 mg.

The total electric charge flowing through the sample during the exposure, which was carried out according to the diagram specified in Table 3, was calculated from the dependence (1).

C = I_1_∙t_1_ + I_2_∙t_2_ + I_3_∙t_3_ + I_4_∙t_4_ [Ah], (1)

where: I_i_—intensity of the polarising current during the next day (A), t_i_—duration of the current flow of a given intensity (h).

The current capacity of alloys was calculated from the Equation (2).

ε = C/∆m [Ah/kg],(2)

where: C—electric charge (Ah), ∆m—mass loss in the tested sample (kg). 

## 4. Results and Discussion

### 4.1. SEM Microscopic Examination for Alloy Samples Prepared for Tests

**Figure 4:**
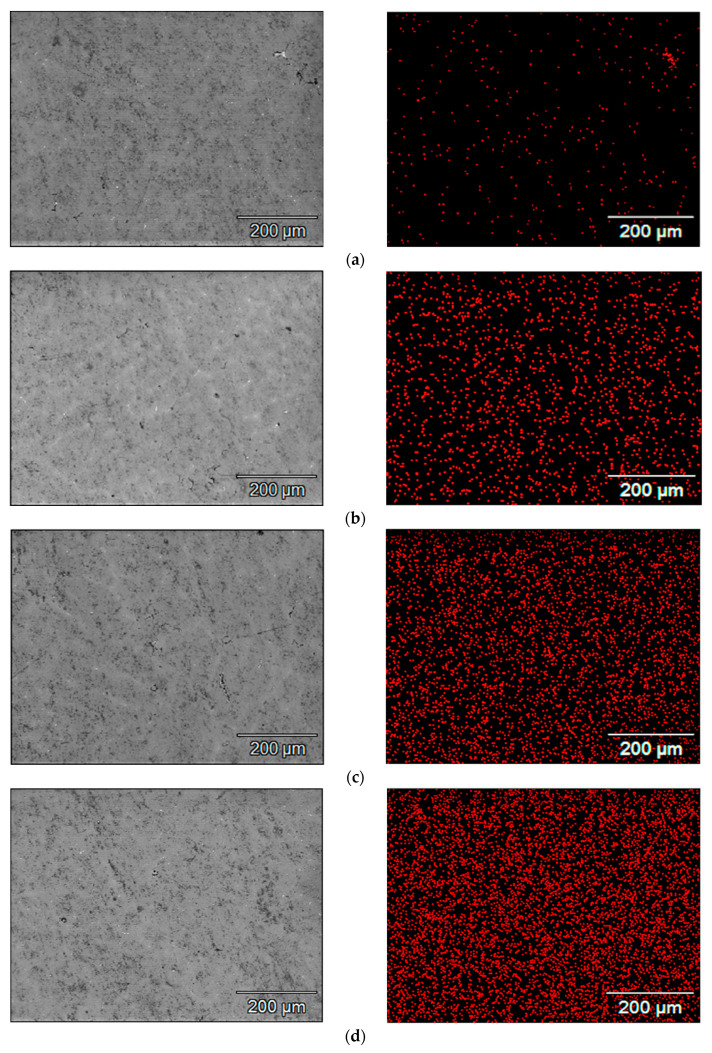

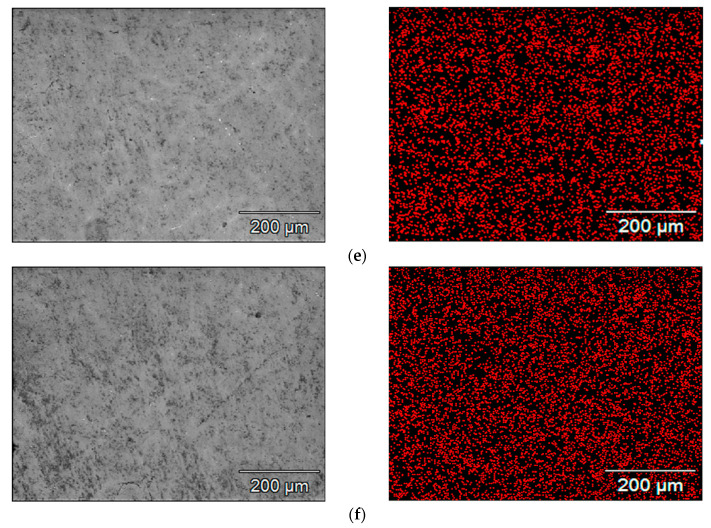
Maps of indium distribution in the tested alloy samples with different indium content, i.e., (**a**) 0.022%; (**b**) 0.034%; (**c**) 0.048%; (**d**) 0.088%; (**e**) 0.133%; (**f**) 0.197%.

SEM images of the microstructure of the samples and the maps of indium concentration and its distribution in the scanning area, which were obtained for individual alloys containing a different amount of indium, are presented in Figure 4. Each red point corresponds to a pulse of X-ray radiation characteristic to indium. The density of the points, which increases in the subsequent Figure 4a–f, corresponds to the increasing concentration of indium in the subsequent samples under tests. It can be seen that the indium is well dissolved in the alloy matrix. It is evenly distributed, there are no separate clusters of this metal in the alloy. 

**Figure 5:**
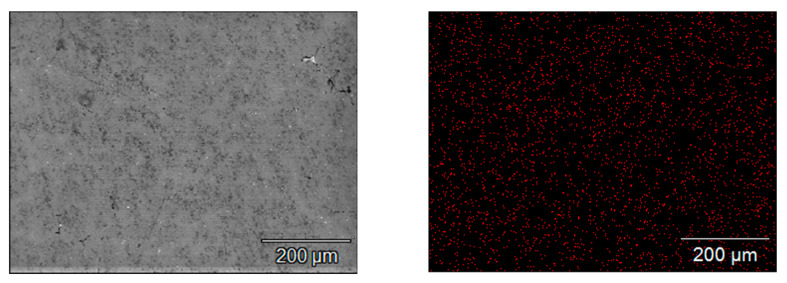
Zinc distribution maps in the base alloy.

A distribution map for zinc atoms in the base alloy (containing 4.24% Zn and 0.022% In) is presented in Figure 5. It is clearly visible that zinc atoms are evenly distributed in the alloy, there are no clusters of this element in the alloy matrix.

### 4.2. Electrochemical Studies of Alloys

The determined current capacity values for the tested alloys are presented in Table 4. The alloy with 0.022% In content had the highest capacity amounting to approx. 2504 Ah/kg. The alloy current capacity decreases as the indium content increases. With a content of approx. 0.05% In, the capacity was reduced to approx. 2350 Ah/kg. The alloy with an indium content close to 0.2% had a capacity as much as 30% lower than the base alloy and it only amounted to approx. 1750 Ah/kg. Table 4 also shows the consumption values for the anode alloy mass, which are reciprocal to the current capacity converted to a yearly value. This consumption demonstrates the mass loss of the anode material during the year, with the anode’s dissolving current flow having the intensity of 1 A. It can be seen that for 0.2% In content the material consumption is as high as 5 kg/(A∙year), when compared to 3.5 kg/(A∙year) for an alloy containing 0.02% In.

**Figure 6:**
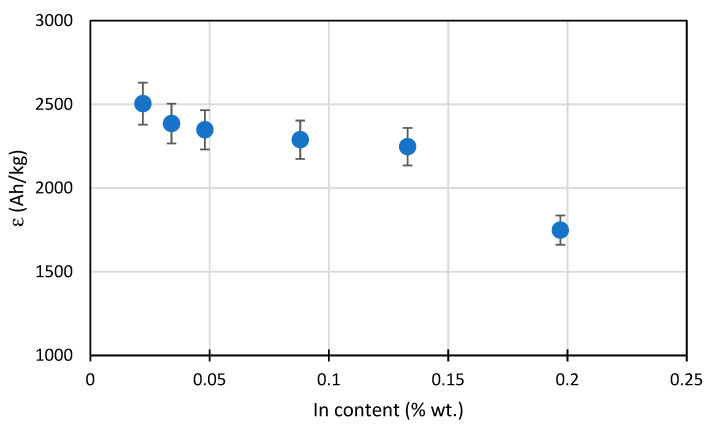
Diagram for the mean current capacity of Al-Zn-In alloys depending on the In content.

In Figure 6, illustrating the changes in the current capacity of the tested alloys, a sharp capacity drop is visible when the indium content reaches 0.2%. In the range of 0.05–0.15% In, the capacity drop is moderate. It can be stated that the alloys with the maximum 0.05% In content are practically applicable for cathodic protection in seawater. For comparison: in work [21] the optimum concentration 0.02% In was found when zinc content was 5%, and in work [29] 0.015% In and 3.5% Zn, respectively.

Values of the potentials of the tested samples are presented in Table 5. In this table, the OCP potential values (before starting the polarisation) and the CCP potentials of the polarised samples at the end of each experiment phase, as well as the density of the polarising current flowing out from the samples in each phase (day), are presented. As the indium content increases from 0.02 to 0.05%, the OCP potential value is more negative by about 50 mV, which is advantageous for using this alloy as a sacrificial anode. A further increase in the indium content no longer changed the OCP potential of the alloy. During polarisation of alloys, a particular dependence can be clearly seen, namely the higher density of the current flowing out from the sample, the more positive value of the CCP potential. With a very high current load for the anode on the third day of exposure, the potential of the polarised samples was more positive than the OCP potential, by as much as 200 mV.

**Figure 7:**
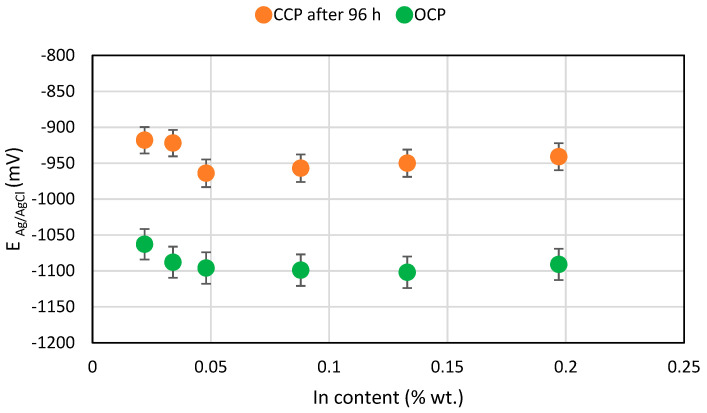
Diagram for the mean OCP and mean CCP potentials for the tested Al-Zn-In alloys in relation to the In content.

According to the adopted experiment procedure in accordance with DNVGL-RP-B401 [30], the main parameter considered when it comes to using the tested alloy as a sacrificial anode for offshore structure cathodic protection is the sample’s potential at the end of the exposure (after 96 h of polarisation). Figure 7 shows the OCP and CCP potential values at the end of the exposure. The alloy with 0.048% In content had the most negative CCP value. The alloys with 0.05–0.20% indium content have similar CCP potential values and they are noticeably more negative (by 30–40 mV) than alloys with 0.02–0.04% content.

### 4.3. Post-Exposure Surface of the Samples

**Figure 8:**
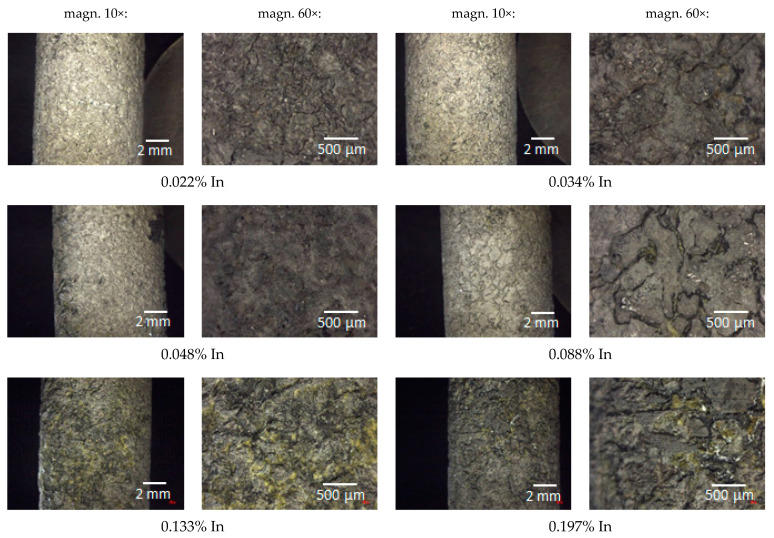
Post-exposure surface views of the tested alloys.

The pictures in Figure 8 illustrate the surface view of the tested anode alloys after the exposure, at 10× and 60× magnification. 

Figure 8 demonstrates that corrosion due to dissolution of the alloy by the polarising current was general in nature and took place over the entire surface of the samples. However, the surface morphology of individual samples is diversified. As the indium content increases, a generally uniform corrosion pattern changed more and more to an uneven one. The size of local metal cavities (pits) increased, and they became longer and deeper. Local cavities on the sample surface were the smallest in the case of the sample containing 0.022% In, and the largest cavities were observed on the surface of the samples with the 0.133% and 0.197% In content.

**Figure 9:**
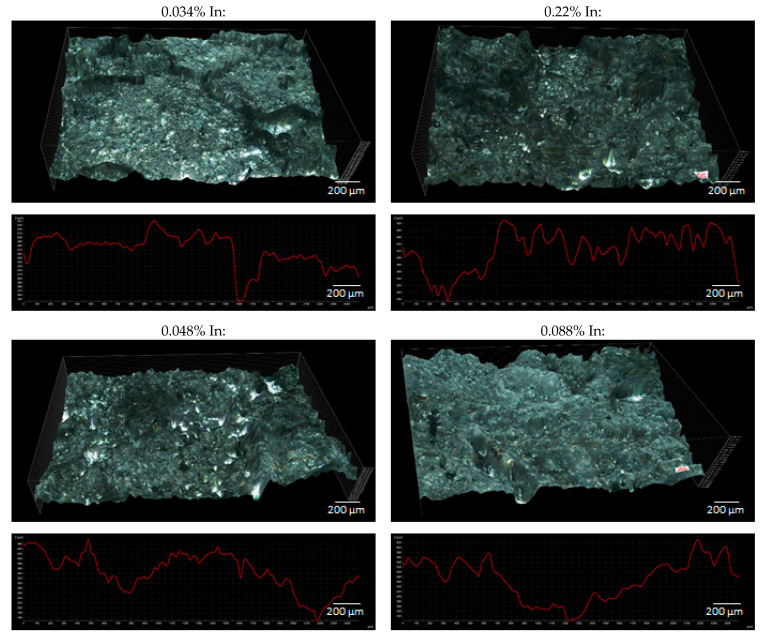

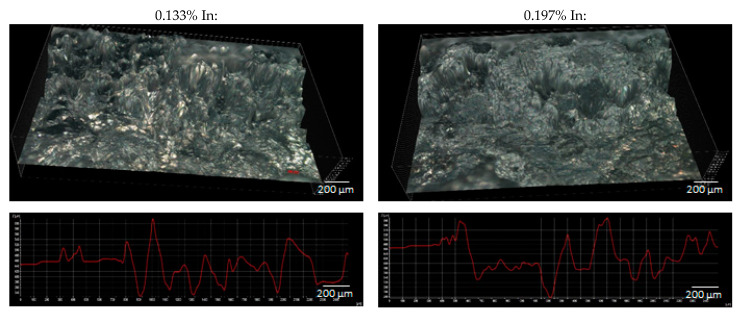
3D view and surface profiles of the tested alloys after the exposure.

Figure 9 shows the 3D surface images for the samples after the exposure and the surface height profiles obtained along the X-axis in the central part of the 3D views. Based on the diagrams of the height profiles, the surface roughness coefficient R_a_ was calculated for each sample, according to the formula (3). The roughness coefficient values for individual samples are presented in Table 6.

R_a_ = (1/n)∙Σy_i_ (3)

where: y_i_—deviation of the ordinate value of the ith measurement point from the mean ordinate value of all the points that make up the profile plot, n—number of measurement points.

The y_i_ are absolute values of differences between the ordinates that make up the profile plot and the mean value of all ordinates. The R_a_ value reflects the size of the cavities (pits) on the surface of the tested samples. So, a value of the roughness coefficient calculated according to the Formula (3) can be considered as a measure of the size of the defects on the surface of the samples. The higher the R_a_ value, the greater the pitting depths on the sample surface, and therefore the greater the weight loss of the tested alloy—and shorter operation time of sacrificial anodes and the lower their current capacity.

The obtained roughness coefficient values confirm the visual assessment described on the basis of Figure 8, which pertains to the increasing unevenness and depth of corrosion defects on the sample surfaces with an increasing In content. A higher corrosion rate (greater weight loss) may result from the fact that a passive oxide layer is more easily formed on the surface of alloys with higher indium content, which then breaks and local dissolution of Al-Zn-In alloy occurs [33]. The increased mass loss induces the reduced current capacity of the alloy.

## 5. Discussion

The electrochemical properties of the individual components of the Al-Zn-In alloy differ significantly. According to the voltage series of metals (giving the potential values versus the standard hydrogen electrode), the lowest potential is revealed by aluminum −1.66 V, then zinc −0.76 V and indium −0.33 V. Therefore, corrosive galvanic cells are formed on the surface of the alloy in contact with the electrolyte, that arise between micro-regions containing metals or intermetallic alloys with different potentials. In these cells, Al is the dissolving anode, and Zn and In are the cathode. The anode-cathode potential difference in Al-In microcells is very large, so the intensity of the corrosion current is also high. This means a high corrosion rate in these microcells.

**Figure 10:**
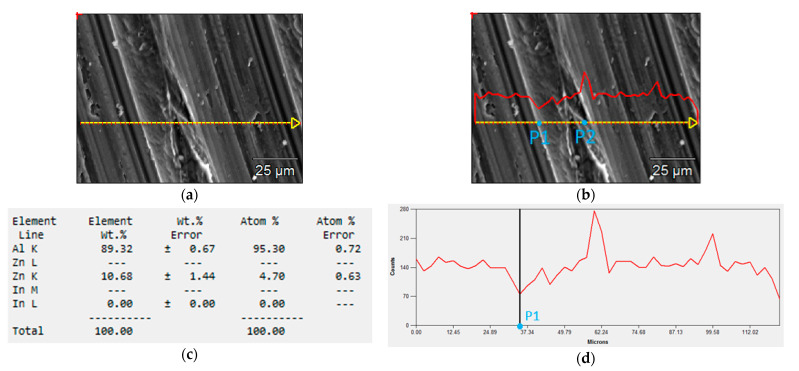

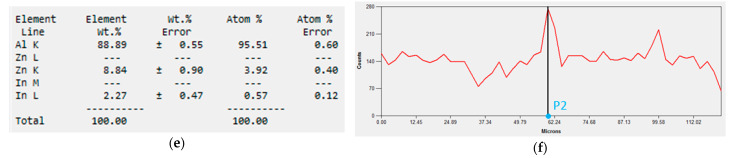
SEM tests of the local content of In in the alloy: (**a**) measurement section, (**b**) changes in the amount of In along the measurement section (red line), (**c**) local element content of the alloy at the point P1, (**d**) graph of the indium content along the measurement section with the location of the point P1, (**e**) local element content of the alloy at the point P2, (**f**) graph of the indium content along the measurement section with the location of the point P2.

Figure 10 shows the result of the SEM test for indium content at various locations in the alloy. The tests were carried out on the sample No. 6 with a content 0.197% In in the whole alloy. The measurements were taken at 50 points along the section marked in yellow in Figure 10a. The graph of indium content along this section is shown in the red line in Figure 10b,d,f. 

There is a quantitative differentiation of the In content in particular places on the tested surface of the sample. Its amount at the point P1 was about 0% (Figure 10c), while at the point P2 it was as much as 2.27% (Figure 10e). This means that after the alloy is immersed in an electrolyte, galvanic microcells will be formed near the point P2 and a greater weight loss of the alloy will occur compared to places with lower indium content.

When comparing alloys with different indium content, the increasing amount of this element contributes to the increased activity of corrosion microcells in the alloy. Thus, the corrosion susceptibility of the alloy increases with increasing indium content. 

Additionally, electrolyte alkalisation takes place around the cathodes in the corrosion cells (OH^−^ ions generated as a result of the oxygen reduction reaction at the cathode). Such an electrolyte may accelerate the corrosion of the alloy matrix, as aluminum is not resistant to the alkaline environment.

In the research presented in this work, the intensity of the corrosion processes described above in the microcells was enhanced by the current polarizing anodically the samples during exposure, which also causes the dissolution of the alloy. Therefore, along with the increase in indium content, the corrosion losses (pitting on the surface of the samples) were greater and greater and the weight loss of the samples increased.

Figure 6 shows that a rapid increase in the intensity of the corrosion processes of the working sacrificial anode occurs when the amount of indium in the Al-Zn-In alloy is above 0.15%. The effect of this is a reduction in the current capacity of such an alloy. 

## 6. Conclusions

The obtained test results demonstrate that Al-Zn-In alloys with an In content of 0.05–0.15% can be successfully used as sacrificial anodes in these applications and cases, in which the desired anode potential value should be lower by several dozen mV. However, the service life of such anodes is reduced by 5–10% (operating time until the complete dissolution of the anode), when compared to anodes with 0.02% In content.

Increasing the In content above 0.15% has a very negative effect on the current capacity of Al-Zn-In alloy, which is significantly decreased in such a case. For 0.2% In content, the current capacity is lower by as much as 30%, in comparison to the alloy with 0.02% In, and the consumption of material for this sacrificial anode increases dramatically and amounts to as much as approx. 5 kg/(A∙year), in comparison to 3.5 kg for 0.02% In content. 

With consideration of all the determined electrochemical parameters of the tested alloys (i.e., the potential of the operating anode, its current capacity and consumption rate for the material), the economic aspects (i.e., anode service time and the requirement for its replacement with a new one), as well as the environmental aspects (i.e., contamination with the corrosion by-products after dissolution of the anode), it can be stated that the Al-Zn-In alloys with no more than 0.05% In content are practically applicable for cathodic protection in seawater.

## Figures and Tables

**Table 1:** The percentage content of the elements in the tested Al-Zn-In alloys (% by weight).

Alloy Number	Zn	In	Al
1	4.24	0.022	balance
2	4.19	0.034	balance
3	4.12	0.048	balance
4	4.22	0.088	balance
5	4.12	0.133	balance
6	4.17	0.197	balance

**Table 2:** The amount of reagents per 10 dm^3^ of tap water, in order to prepare the synthetic seawater.

Reagent	Weight (g)
NaCl	245.34
Na_2_SO_4_	40.94
MgCl_2_·6H_2_O	111.24
CaCl_2_	11.58
SrCl_2_·6H_2_O	0.42
KCl	6.95
NaHCO_3_	2.01

**Table 3:** Density of the current flowing from the samples during the experiment.

day one:	j_1_ = 1.5 mA/cm^2^
day two:	j_2_ = 0.4 mA/cm^2^
day three:	j_3_ = 4.0 mA/cm^2^
day four:	j_4_ = 1.5 mA/cm^2^

**Table 4:** Mean current capacity and mean annual mass consumption for the tested Al-Zn-In anode alloys.

Alloy Number	In Content (%)	Current Capacity (Ah/kg)	Consumption of Anode Material (kg/(A∙Year))
1	0.022	2503.98	3.48
2	0.034	2381.22	3.54
3	0.048	2347.54	3.75
4	0.088	2290.13	3.82
5	0.133	2247.13	3.88
6	0.197	1749.35	5.03

**Table 5:** Mean open circuit potential (OCP) and mean closed circuit potentials (CCP) of the tested alloys vs. Ag/AgCl/seawater electrode after the next exposure stages.

Alloy Number	In Content (%)	OCP (mV)	CCP (mV)			
			After 24 h j = 1.5 mA/cm^2^	After 48 h j = 0.4 mA/cm^2^	After 72 h j = 4.0 mA/cm^2^	After 96 h j = 1.5 mA/cm^2^
1	0.022	−1065	−956	−935	−881	−919
2	0.034	−1079	−968	−908	−895	−922
3	0.048	−1099	−988	−919	−968	−970
4	0.088	−1100	−1045	−963	−930	−963
5	0.133	−1108	−1013	−938	−942	−945
6	0.197	−1099	−986	−969	−912	−937

**Table 6:** Surface roughness coefficient of the tested anode alloy samples after the exposure.

Alloy Number	In Content (%)	Roughness Coefficient R_a_
1	0.022	35.25 µm
2	0.034	44.92 µm
3	0.048	62.99 µm
4	0.088	72.34 µm
5	0.133	91.78 µm
6	0.197	94.62 µm

## Data Availability

Data sharing is not applicable to this article.
